# Combination of a Proteomics Approach and Reengineering of Meso Scale Network Models for Prediction of Mode-of-Action for Tyrosine Kinase Inhibitors

**DOI:** 10.1371/journal.pone.0053668

**Published:** 2013-01-09

**Authors:** Stefan Balabanov, Thomas Wilhelm, Simone Venz, Gunhild Keller, Christian Scharf, Heike Pospisil, Melanie Braig, Christine Barett, Carsten Bokemeyer, Reinhard Walther, Tim H. Brümmendorf, Andreas Schuppert

**Affiliations:** 1 Department of Oncology, Haematology and Bone Marrow Transplantation with Section Pneumology, Hubertus Wald-Tumor Zentrum (UCCH), University Hospital Eppendorf (UKE), Hamburg, Germany; 2 Division of Hematology, University Hospital Zürich, Zürich, Switzerland; 3 Department of Biochemistry, University Hospital Aachen (UKA) of the Rheinisch.-Westfälische Technische Hochschule Aachen, Aachen, Germany; 4 Department of Medical Biochemistry and Molecular Biology, University of Greifswald, Greifswald, Germany; 5 Interfacultary Institute of Genetics and Functional Genomics, University of Greifswald, Greifswald, Germany; 6 Department of Otorhinolaryngology, Head and Neck Surgery, University of Greifswald, Greifswald, Germany; 7 Bioinformatics, University of Applied Sciences Wildau, Wildau, Germany; 8 Medizinische Klinik IV - Hämatologie und Onkologie, Universitätsklinikum Aachen (UKA) of the Rheinisch.-Westfälische Technische Hochschule Aachen, Aachen, Germany; 9 Aachen Institute for Advanced Study in Computational Engineering Science (AICES), RWTH Aachen University, Aachen, Germany; German Cancer Research Center, Germany

## Abstract

In drug discovery, the characterisation of the precise modes of action (MoA) and of unwanted off-target effects of novel molecularly targeted compounds is of highest relevance. Recent approaches for identification of MoA have employed various techniques for modeling of well defined signaling pathways including structural information, changes in phenotypic behavior of cells and gene expression patterns after drug treatment. However, efficient approaches focusing on proteome wide data for the identification of MoA including interference with mutations are underrepresented. As mutations are key drivers of drug resistance in molecularly targeted tumor therapies, efficient analysis and modeling of downstream effects of mutations on drug MoA is a key to efficient development of improved targeted anti-cancer drugs. Here we present a combination of a global proteome analysis, reengineering of network models and integration of apoptosis data used to infer the mode-of-action of various tyrosine kinase inhibitors (TKIs) in chronic myeloid leukemia (CML) cell lines expressing wild type as well as TKI resistance conferring mutants of BCR-ABL. The inferred network models provide a tool to predict the main MoA of drugs as well as to grouping of drugs with known similar kinase inhibitory activity patterns in comparison to drugs with an additional MoA. We believe that our direct network reconstruction approach, demonstrated on proteomics data, can provide a complementary method to the established network reconstruction approaches for the preclinical modeling of the MoA of various types of targeted drugs in cancer treatment. Hence it may contribute to the more precise prediction of clinically relevant on- and off-target effects of TKIs.

## Introduction

Tyrosine kinase inhibitors (TKIs) are nowadays frequently used for treatment of defined solid and hematological cancer entities. Although these drugs are typically developed for the targeting of single kinases which are specifically overexpressed in cancer cells [1], [2], [3], in reality they usually inhibit a multitude of kinases and nonkinase targets [4], [5], [6], [7] resulting in a heterogeneous activity profile which is poorly predictable. Based on this off-target activity most of the clinically used TKIs exert relevant side effects which can interfere with the efficacy of the treatment regime [8], [9], [10] leading to unfavorable therapeutic windows. Therefore, the prediction of drug action profile as early as possible in the drug research and discovery process is of eminent importance to avoid clinical trials using compounds with unforeseen unfavorable efficacy – risk profiles. The realization of the “fail early principle”, however, requires methods to extract drug action from drug response profiles based on high throughput testing in well defined cell culture systems. Moreover, identification of the full set of modes-of-action (MoA) of drugs and the assessment of their respective impact on secondary drug action are of utmost importance both for optimal selection of targets or alternatively, combinations of targets for optimization of future drug discovery as well as for the optimal administration of already existing compounds. Due to the molecular complexity of the various cancer entities, network reconstruction of MoA from combinatorial drug experimentation will be of special relevance for cancer therapies [11]. Several methods for identification of MoA, side effects and drug efficacy from cellular drug responses have been described. Prediction of drug efficacy as well as potential adverse side effects can be performed by chemical structures and experimental data from cell screening experiments of the compounds using appropriate similarity scores [12], [13], [14], [15], [16]. An alternative approach uses established network information with respect to known MoA’s and predicts side effects identified by cooperative pathway analysis [17]. Experimentally derived dose-response surfaces from combinatorial drug experiments can be used to identify simplified or detailed models for the respective MoA’s and their interactions from analysis of the combinatorial drug response surfaces [18], [19], [20]. The reconstruction is performed by a systematic fit of models for drug action to the dose-response surfaces, whereas the underlying models can show a widely varying degree of detail. The models can be based on the simplified concepts of Loewe additivity and Bliss independence and go up to mechanistic systems biology models, where the respective pathways involved in the MoA are represented in detail and have to be fit to the data. However, due to the lack of data and detailed understanding of the MoA, model fitting from dose-response surfaces may become ill-posed when the grade of details represented by the model is increased. Hence, model-fitting approaches tend to result in ambiguous network reconstructions when the size of the networks becomes large. The ill-posedness can be reduced by reduction of complexity either by shrinking the models to simplified network topology or by reducing the interaction between involved pathways to simplified mechanisms, such as boolean networks. In any case there will be payoffs by loosing biological features which are specific to the model. Hence, most applications tend to analyse the data using a set of models and decide according to a ranking of the respective model accuracies. A more generic drawback of fitting models to drug-response surfaces arises when the MoA’s are not fully understood. Then the inherent issue arises that the model structure does not represent the biological mechanisms, which can lead to systematic errors in network reconstruction and model predictivity. Another approach to overcome the ill-posedness of models derived from combinatorial drug-response surfaces may be a systematic integration of multiple outputs of drug action into a network integrating drug descriptors, MoA and pharmacological data [21]. Whereas the abovementioned approaches focus on specific pharmacological applications, combinatorial network reconstruction has been used to reconstruct generic signaling networks as well. Most of these approaches are based on evaluation of drug treatment on gene expression or protein phosphorylation profiles and the subsequent development of algorithms for reengineering of signaling networks [22], [23]. Combinatorial optimization algorithms are mostly being used in order to identify the relevant signaling networks out of a given set of pathway proteins. In principal, these networks have the potential to enable the identification of direct and specific drug targets or preferentially affected signaling pathways [24], [25]. Recently efficient, systematic and direct network reconstructions of induced phosphorylation of signaling proteins have been reported using combined stimulation and inhibition of cell cultures [26], where complex interaction networks have been reconstructed in detail from data describing combinatorial stimulation and inhibition of cells, using a highly multi-variate readout (phosphorylation of signaling proteins). Despite the tremendous improvement of understanding complex signaling networks and the interaction of the relevant pathways, drug effects mediated by yet unexpected cellular mechanisms, potentially as a secondary response on the primary drug action, may not sufficiently be assessed due to lacks in model structures. Hence novel unsupervised network reconstruction algorithms which are based on data obtained from broad-scale transcriptome and/or proteome profiling are needed as complementary method.

In this paper we use a combinatorial network reengineering approach which is based on data representing the combinatorial effect of multiple input data (TKI’s and mutations) on multiple output data (set of proteins responding on the combinations of administered drugs and mutations).

The respective analysis is of very high relevance to targeted therapies, where development and/or selection of mutations in the targets or in the addressed pathways plays a major role in drug resistance with high relevance for personalized therapeutic approaches. In this case the drug-response surface is not continuous, since the mutations (as a combinatorial input variable) induce a discrete structure in the inputs, hampering the application of fitting of models from drug-response surfaces. Moreover, the screening was performed only for four drugs, which are known to show specific action against the target, in one concentration only, so the broad data set required for unsupervised approaches was not available and models based on chemical structures leading to the prediction of broad side effects will not be specific enough. In addition, due to the unspecific targeting of thyrosine kinases by TKI’s we aimed to assess the MoA on a proteome-wide scale.

**Figure 1:**
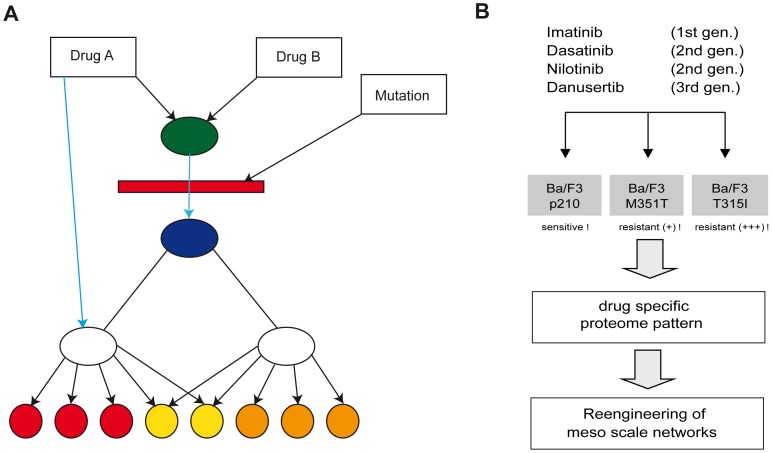
Schematic representation of meso network architecture and experimental design. (**A**) Exemplifies an abstract meso-scale network representing (abstract) pathways of drug action of drugs A and B on induction of proteins (red, yellow and orange bullets). Drug A uses two pathways (blue and black), whereas the blue pathway induces expression shifts only on a subset of the proteins (red, yellow), whereas the black pathway induces expression shifts in all proteins. Drug B acts only via one pathway which joins the black pathway of Drug A in an abstract node (represented by the green bullet). The mutation inhibits both pathways between the green and blue bullet resulting in an interference with drug induced expression shift for all proteins. The blue pathway from Drug A to the proteins, however, is not affected by the mutation. Hence the mutation may have a strong impact on the efficacy of drug B, whereas the profile of action of drug A is only altered by the mutation. (B) Imatinib sensitive and resistant cells were treated with four tyrosine kinase inhibitor and mesoscale network were reengineered based on specific proteome expression patterns.

Because of the sparse data available, we used a direct network reconstruction approach which is focused on the identification of unknown network topologies on a simplified level of details [27]. Similar to the approach used in [17], the model describing the mechanisms of interaction between the input variables (here drugs and mutations) and the output variables (here induced protein expression and apoptosis) is represented by an abstract network. In contrast to network models representing the detailed mechanisms (where the nodes may represent explicitly addressed proteins or genes), our abstract network reconstruction identifies only (abstract) pathways linking drugs and the readouts (here protein expression and apoptosis), the overlap of the pathways as well as the localization of the pathway disruption by mutations (Figure 1A). The edges represent the induction of a biological effect (either activation or inhibition) by the drug, whereas the nodes represent junctions of the pathways or breakpoints where a pathway can be interrupted by a mutation. For simplicity, the breakpoints where a pathway can be interrupted by a mutation can be represented by a node located on an edge, too (red bar). Although the nodes in the abstract network model do not represent well identified biological mechanisms, the model provides an overview about the existence of multiple pathways controlling the drug action as well as their mutual interactions and interaction with mutations. As the model can be identified in an unsupervised manner, it may provide a first stage towards more detailed modeling helping to avoid a bias due to incomplete a priori knowledge. Moreover, this concept allows a continuous transition of model types in terms of the level of details, starting from very black-box models ending up at fully mechanistic models [28] and is established in modeling complex chemical processes [29], [30]. Because of the variable level of network details, we call this type of models meso-scale networks. Recently it has been shown, that the topology of such meso-scale networks can be reconstructed from data without fitting of models, even if almost no restriction is assumed for the functional form of the nodes [27]. The drawback is that the method so far requires a feed-forward structure which can be decomposed into tree structures, so no feedback loops can be identified. Hence the method is mostly restricted on network reconstruction on a rough level of details. Although this approach does not directly specify the role of specific biological mechanisms in drug response, it allows the assessment of the number of pathways and their interaction providing hypothesis for detailed mechanistic follow-up research. It thereby provides a valuable approach which is complementary to the established methods providing an unbiased assessment of mechanisms and their interaction on a proteome-wide scale in a first level analysis.

Chronic myeloid leukemia (CML) represents an excellent model disease for development of cancer-specific TKIs [31]. Currently, three different TKIs, Imatinib (IM) [32], Nilotinib (NILO) [33] and Dasatinib (DASA) [34], are approved for first and second line treatment and novel drugs such as dual BCR-ABL/src inhibitor Bosutinib [35], [36], [37], dual aurora/BCR-ABL inhibitor Danusertib (DANU) [38] and multi targeted TKI Ponatinib are being evaluated in clinical trials [39], [40], [41]. From clinical use and experimental evidence it is known that the efficacy and side-effect profile of individual TKIs depends on targeted MoA as well as on indirect responses based on the unspecific inhibition of various kinases [1].

In the current study, we aimed to develop a model approach for the prediction of the MoA of TKIs reengineered from global proteome data. We describe unraveling of meso scale networks based on protein expression changes induced by four kinase inhibitors (IM, DASA, NILO and DANU) in wild type BCR-ABL positive cells as well as in mutants (i.e. BCR-ABL mutation M351T and T315I) which confer different degrees of resistance to the TKIs tested. Furthermore, we present a model for integration of protein expression data and induction of apoptosis. In conclusion, we present a promising novel approach which can be used for prediction of multiple drug MoA in various clinical settings. We show that screening of protein response on combinatorial stimulation by drugs as well as inhibition by mutations can be used for the delineation of the mechanisms of drug resistance.

## Results

### Effects of TKIs on the Induction of Apoptosis in BCR-ABL + and − Cells

**Figure 2:**
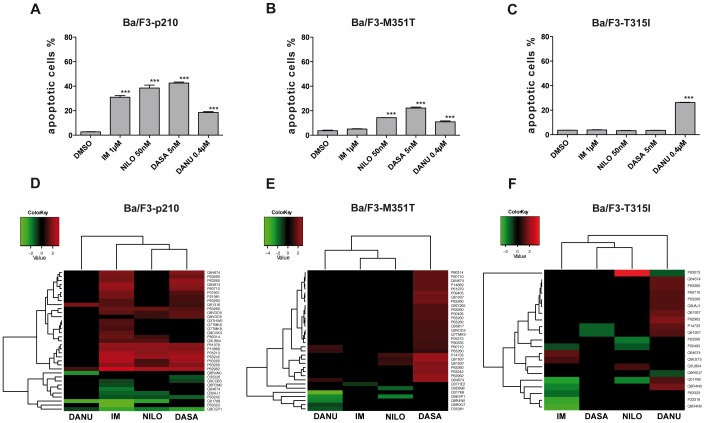
A–F: Apotosis induction after treatment with different TKI and hierarchical clustering of differential expressed protein. (**A**) Ba/F3-wt. –p210, -M351T und –T315I cells were incubated with Imatinib (IM), Nilotinib (NILO), Dasatinib (DASA) or Danusertib (DANU) for 24 hours. After 2D-PAGE changes in the protein expression profile was analyzed using Delta-2D software to identify drug-specific. (**B–E**) Caspase 3 activity in BCR-ABL negative Ba/F3 cells (B), wildtype IM sensitive BCR-ABL positive Ba/F3-p210 cells (C) and mutated IM resistant BCR-ABL positive Ba/F3-M351T-(D) and BAF3/−T315I cells. Asterisks indicate significant changes compared to DMSO. Unsupervised clustering (euclidean distance measure and the 'average' agglomeration method) was performed using the log transformed expression protein values for (**D**) Ba/F3-p210, (**E**) Ba/F3-M351T and (**F**) Ba/F3-T351I cells. The samples are shown horizontally, the proteins vertically. The dendrograms represent the distances between the clusters. In the upper color bar, the upregulated proteins are marked in red, the down regulated are shown in green.

For the in vitro screening of TKI-dependent proteome changes, we used a well-established murine CML model: Ba/F3 cells, an immortalized murine bone marrow-derived pro-B-cell line, are retroviraly transfected with either wild type (p210) or mutated BCR-ABL isoforms (M351T, T315I). In order to compare the efficacy of the different TKIs, concentrations with similar inhibitory activity (close to the respective IC50s of the TKIs) were used (Figure 1B). Induction of apoptosis served as an indicator for efficient inhibition of BCR-ABL activity and was measured by caspase-3 activity in flow cytometry. Figures 2A–C show a comparable degree of induction of apoptosis in TKI sensitive cells indicating a successful inhibition of BCR-ABL. Consequently, a distinct response pattern was observed by treatment with TKI of the first (IM), second (NILO, DASA) and third generation (DANU) in BCR-ABL positive Ba/F3-p210 cells (Figure 2A) while no apoptosis was observed in BCR-ABL negative Ba/F3 control cells (not shown). Expectedly, in cells harboring the low-level IM resistance conferring M351T mutation, DASA and NILO were found to be active (Figure 2B). However, in cells transfected with the highly resistant T315I mutant, only the third generation inhibitor DANU, a dual inhibitor of BCR-ABL and aurora kinases A, B and C, was capable of induction of apoptosis. As expected the efficacy of DANU was slightly more pronounced in the T315I mutant as compared to the wild type form of BCR-ABL (Figure 2C). This effect is based on particular structural properties of DANU which allows binding to the active center of the mutant kinase [42].

### Identification of Differentially Regulated Proteins in BCR-ABL+ cells Treated with TKIs

Sets of independent triplicates for each BCR-ABL isoform treated for 24 hours with the different TKIs were analyzed and compared to the solvent control (DMSO) by two-dimensional gel electrophoresis (2D-PAGE) analysis. As expected and in line with the reported alterations of caspase-3 activity, a wide response to the TKIs was observed in wt Ba/F3-p210 cells. In total, 68 spots with a differential expression of at least 2-fold and statistical significance were identified (Table S1). For each BCR-ABL isoform a specific drug profile as well as a proteome map with the individually regulated spots was generated. Thus, in Ba/F3 cells harboring the wt BCR-ABL, 46 (35 up and 11 down), 24 (20 up and 4 down), 34 (28 up and 6 down) and 10 (6 up and 4 down) specific spots were detected secondary to treatment with IM, NILO, DASA or DANU, respectively.

In Ba/F3 cells harboring the low grade IM resistant mutation M351T, IM changed the expression of one spot (0 up and 1 down) while NILO altered the expression of 5 spots (3 up and 2 down), DASA of 28 spots (28 up and 0 down) and DANU of 8 spots (3 up and 5 down) (Table S2). As expected, alterations reported in Ba/F3 cells transfected with the highly resistant T315I mutation were minor. Thus, only eight altered spots (2 up and 6 down) were reported in IM, five (2 up and 3 down) in NILO and two (0 up and 2 down) in DASA treated cells. However, in DANU treated cells 14 spots (12 up and 2 down) were found to have changed expression (Table S3). Secondary to the identification of the quantitatively altered protein spots by Delta-2D software a characterization by mass spectrometry was performed.

### TKI Specific Effects on Protein Expression in IM Sensitive and IM Resistant Ba/F3 Cell Lines

To analyze a potential correlation between the level of resistance to the respective TKIs and a similar protein expression pattern, cluster analyses were performed (Figure 2D–F). Remarkably, hierarchical cluster analysis based on these candidate proteins identified similar protein expression patterns for IM, NILO and DASA when compared to DANU in Ba/F3-p210 cells (Figure 2D). However, in TKI resistant cell lines, clustering was less pronounced (Figure 2E–F). The evaluation of changes of the protein expression patterns depending on the applied drug and the BCR-ABL mutation status allows us to describe more closely on target and off target nature of effects of the respective TKIs. Furthermore, the comparison of the protein expression patterns of the different TKIs with known off-target activity (such as the SRC family of kinases in the case of DASA of the Aurora kinases in the case of DANU) in wt BCR-ABL positive Ba/F3-p210 cells allows to characterize protein expression changes observed as a result of inhibition of BCR-ABL as opposed to changes attributable to inhibition of off target kinases.

**Figure 3:**
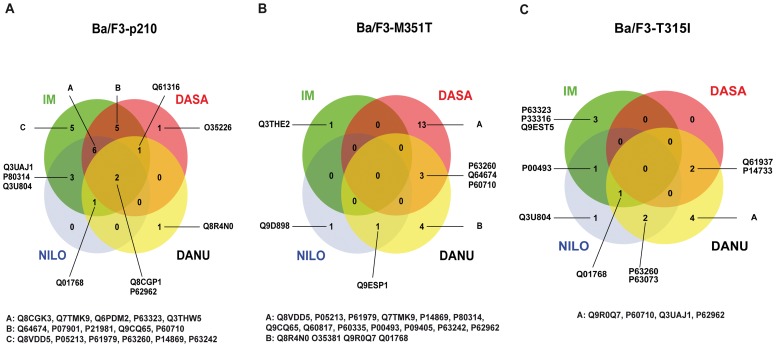
A–C: Venn diagrams for representation of drug specific protein expression. Venn diagrams illustrated the drug specific effects in different cell lines: (**A**) Ba/F3-p210, (**B**) Ba/F3-M351T and (**C**) Ba/F3-T351I cells. The numbers inside the circles represent the number of regulated proteins.

**Figure 4:**
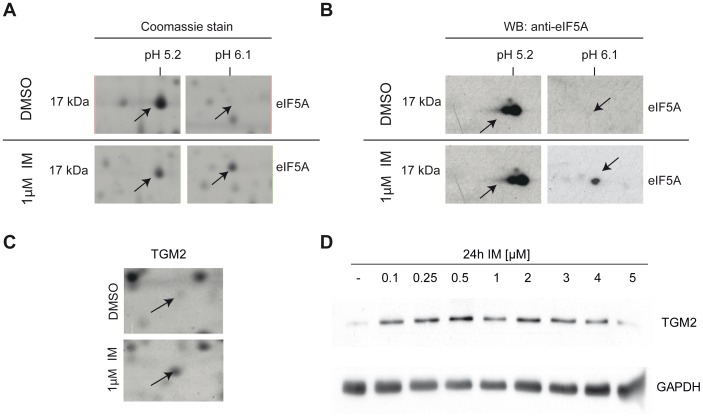
A–D: Western blot analyses revealed posttranslational modification of eIF5A and up regulation of TGM2 after treatment with IM. (**A**) Enlarged regions from a coomassie stained 2D-PAGE from Ba/F3-p210 cells after treatment with IM or DMSO as a control. The arrows indicate two spots for eIF5A, one at pI of 5.2 and the other one at a pI of 6.1. The latter appeared after IM treatment. (**B**) 2D-WB validated the appearance of a second spot for eIF5A at a pI of 6.2 after IM treatment. (**C**) Enlarged regions from a coomassie stained 2D-PAGE from Ba/F3-p210 cells after treatment with IM or DMSO as a control. One spot for TGM2 (arrow) demonstrated an increased expression after IM treatment. (**D**) The increased expression of TGM2 after treatment with rising concentrations of IM could be validated in human Bcr-Abl K562 cells.

Surprisingly, by analyzing overlapping expression patterns of candidate proteins induced by the different TKIs in the individual wild-type and mutant cell lines, we found that in Ba/F3-p210 cells, only two of 45 proteins revealed consistently altered expression characteristics secondary to treatment with all four TKIs (Figure 3A). The highest level of consistency was detected for IM, NILO and DASA implying a similar efficacy profile. However, effects that could be assigned to individual cells specifically were very limited, e.g. secondary to IM only five proteins were identified, one after treatment with DASA or DANU and no specific proteins were found in cells treated with NILO (Figure 3A). No proteins simultaneously regulated by all 4 TKIs were observed in the low-level IM resistant Ba/F3-M351T mutant (Figure 3B) nor in highly resistant Ba/F3-T315I cells (Figure 3C). Strongest drug specific effects were detected in DASA treated Ba/F3-M351T cells with 13 proteins differentially regulated whereas DANU revealed four and both IM and NILO only revealed one compound-specific protein each (Figure 3B). Expectedly, in Ba/F3-T315I cells, DANU revealed the strongest drug specific effect with four compound-specific proteins showing altered expression. Surprisingly, a similar degree of compound-specific effect was observed for IM. However, due to the fact, that T315I is highly IM resistant, these effects were attributed to off target effects. NILO induced one specific change while DASA did not show any specific alterations at all (Figure 3C). In order to verify changes in the expression or modification pattern of two of the identified proteins, we performed Western blot analysis for the eukaryotic initiation factor 5A (eIF5A) and for tissue transglutaminase 2 (TGM2). For eIF5A a two-dimensional Western blot revealed the appearance of one additional spot at a pI of 6.1 after treatment with 1 µM IM for 24 hours. This finding is consistent with the Commassie stained large 2D-gels and can be interpreted as a posttranslational modification of eIF5A, which leads to a shift of the protein to a more basic pI (Figure 4A–B). For TGM2 the Western blot analysis confirmed an IM dependent up regulation of the protein. In the Commassie stained 2D-PAGE (Figure 4C) and in the one-dimensional Western blot (Figure 4D) TGM2 showed a clear upregulation.

### Meso Scale Network Models for Action of TKIs

#### Meso scale networks in BCR-ABL wild-type Ba/F3-p210 cells

**Figure 5:**
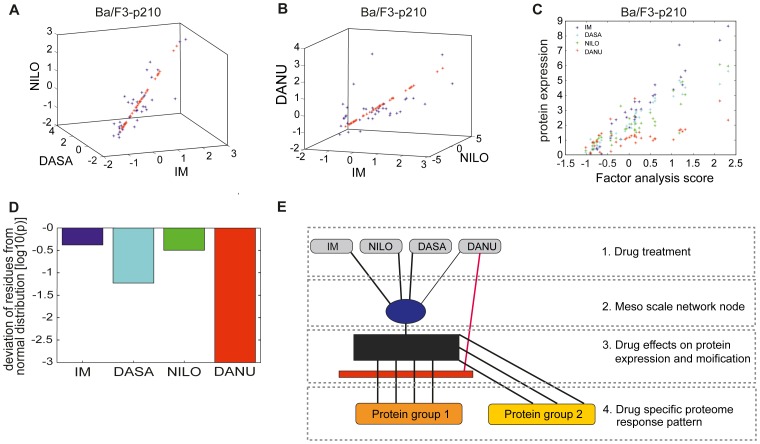
A–E: Meso scale networks Ba/F3-p210 cells. (**A**) High degree of co-regulation across the protein set for IM, DASA and NILO, which can effectively represented by the mean component of factor analysis. (**B**) Significant deviations for a small set of proteins for DANU suggesting the use of the more stable factor analysis instead of PCA for reduction of dimension. (**C**) Analysis quantitatively the amount of induction of protein expression, which is associated with the activation of the dominant mechanisms, quantified by the mean component of factor analysis. Whereas a good and almost similar behaviour for IM, DASA and NILO is observed, DANU activates the proteins in two clearly separated modes (indicated by the upper and lower line of red stars). This finding is supported by quantitatively testing the distribution of the residuals of protein expressions with respect to the linear regression model given by the mean component of factor analysis. (**D**) Apparently only DANU induces residuals with significant non-gaussian noise indicating the existence of two separate mechanisms of protein induction. (**E**) Structure of meso scale pathways for induced protein expression. Black block represents induction of the protein expression by the main pathway, whereas the red block is indicating an inhibition via the main pathway.

**Figure 6:**
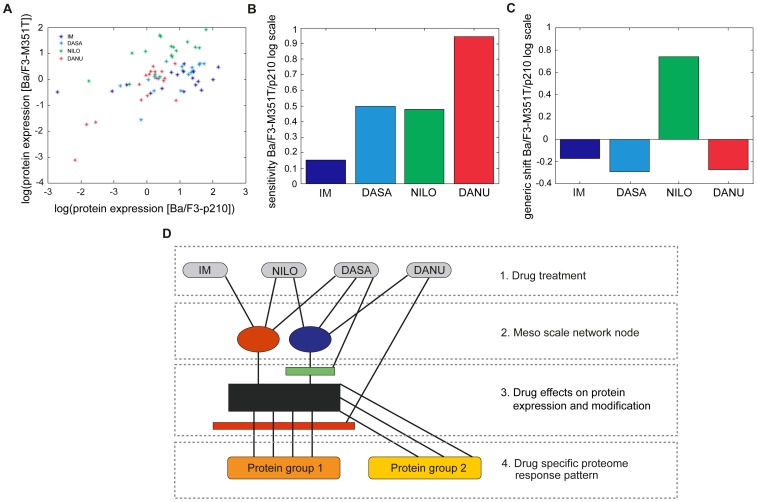
A–D: Meso scale networks Ba/F3-M351T. (**A**) Shows the protein expressions for wt and T351 cell type for all drugs. (**B**) Shows the sensitivity of protein expression with respect to the dominant activation mechanism, quantified by the mean component of factor analysis. (**C**) Shows, that surprisingly the overall level of protein expression induced by NILO increases, although the sensitivity decreases. (**D**) Shows the modifications which are induced by the analysis of the M351I mutation to the meso scale pathway network depicted in Figure 5E. Black block represents induction of the protein expression by the main pathway, whereas the red block is indicating an inhibition via the main pathway. Green block represents the unique effect of NILO on the overall protein expression level.

In wild type Ba/F3-p210 cells, 37 proteins with significant differential expressions were identified. As depicted in Figure 5A, for IM, DASA and NILO, the induced expression changes for all 37 proteins show a high mutual correlation, such that the induced expression for all proteins can be approximated by a joint factor FA (red stars) identified using standard factor analysis. However for DANU, a systematic and significant deviation from the joint expression factor was observed (Figure 5B). This correlated behavior of the 37 proteins is visualized by Figure 5C depicting the protein expressions under all four drugs. IM, DASA and NILO show structurally similar behavior with almost uniform correlation to the factor FA, whereas the response on DANU can be separated into at least two protein groups. The first protein group (group 1, i.e. lower population of red stars, Figure 5C) is correlated to FA, but shows significantly less sensitivity when compared to IM, DASA or NILO, whereas the second protein group (group 2, upper population of red stars, Figure 5C) shows high correlation to FA with high sensitivity. The separation into multiple protein groups with heterogeneous activation by the drug is supported by the analysis of the distribution of the residues of the protein expressions from the factor model. A Lilliefors-test for normal distribution of the deviation has been performed for all four drugs. The respective p-values, depicted in Figure 5D, indicate that the deviations for IM and NILO are normal distributions which are due to noise, whereas the very low p-value for DANU indicates that the respective protein expressions cannot be explained by one factor FA plus random noise alone. For DASA, the respective p-value is slightly higher than 5%, such that a separation into multiple protein groups cannot be excluded. The protein groups for DANU and DASA have been separated using regression clustering (Table S4 for DANU, see supplement). These findings can be interpreted as depicted in Figure 5E. In Ba/F3-p210 wild type cells, IM, DASA and NILO activate pathways which join together in a functional node (blue bullet) which activates all 37 proteins in a coherent manner according to the stimulation of the joint node. In contrast, DANU stimulates the joint node with significantly less impact, (protein group 1), whereas the proteins in group 2 show a similar (or slightly higher) response to stimulation compared to stimulation with IM, DASA or NILO. The black block in Figure 5E (as well as in Figure 6D) indicates the model for induction of the protein expression by the main pathway, in this paper represented by a linear model. The red block in Figure 5E (as well as in Figure 6D) indicates the common inhibition of the drug action for protein group via the main pathway. Hence we propose (at least one) additional component for the mechanism of induction of protein expression by DANU which is depicted in Figure 5E. The findings can be explained if one assumes that DANU activates the joint mechanism similar to the other three drugs, but it induces a second MoA as well. This second MoA reduces the induced expression of the group 1 proteins.

#### Meso scale networks in BCR-ABL mutated BaF/3-M351T cells

The induced protein expressions of 17 proteins both in Ba/F3-M351T cells as well as in Ba/F3-p210 cells are depicted in Figure 6A. Due to the logarithmic scale, induction is represented by positive values and suppressions by negatives. The overall induced protein expressions in BAF/F3-M351T and Ba/F3-p210 cells show a linear correlation on the logarithmic scale, which differs, however, between the various TKI’s. Figure 6B shows the slopes of induced protein expression for the individual TKI’s for Ba/F3-MT351T – cells compared to Ba/F3-p210 wild type cells as calculated from Figure 6A using linear regression. Low values indicate a strong suppression of drug induced protein expression by the mutation, whereas a value around one indicates that the mutation has no significant effect on the MoA’s of the respective drug. Apparently the induction of protein expression by IM is significantly suppressed by the M351T-mutation, whereas the MoA’s of DANU are apparently not effectively inhibited by the M351T-mutation. The MoA’s of DASA and NILO appear to be slightly affected by the mutation. Surprisingly all 17 proteins show a significant, coherent increase of expression in Ba/F3-M351T cells compared to Ba/F3-p210 wild type under NILO treatment (Figure 6C). These findings suggest that the protein expression is controlled by a more detailed mechanism than described above. The joint node controlling the coherent protein expression, as observed in Ba/F3-p210 wild type cells, has to be split into at least two components, one (red bullet) is affected by the M351T-mutation, the other (blue bullet) is not (Figure 6D). IM interacts only with a pathway passing the red node, whereas DASA and NILO affect both nodes. DANU apparently affects only the blue node. Moreover NILO induces the overall expression level (indicated by green bar).

#### Meso scale networks in BCR-ABL mutated BAF/F3-T315I cells

In Ba/F3-T315I-mutated cells compared to Ba/F3-p210 wild type cells, 11 proteins with significant differential expression could be identified (data not shown). The mean expressions under IM and DASA are significantly reduced compared to Ba/F3-p210, whereas the mean logarithmic expressions of NILO and DANU in Ba/F3-T315I cells do not significantly differ from the mean expressions in Ba/F3-p210 wild type. Apparently, in contrast to M351T-mutated cells, no correlation of the expression under T315I-mutation and wild type p210 can be found, hence linear regression analysis is not applicable for analysis of the T315I-mutation. This finding indicates that the T315I mutation appears to interrupt the edge between both control nodes to the protein expression.

### Meso Scale Network Models for Apoptosis Induction

**Figure 7:**
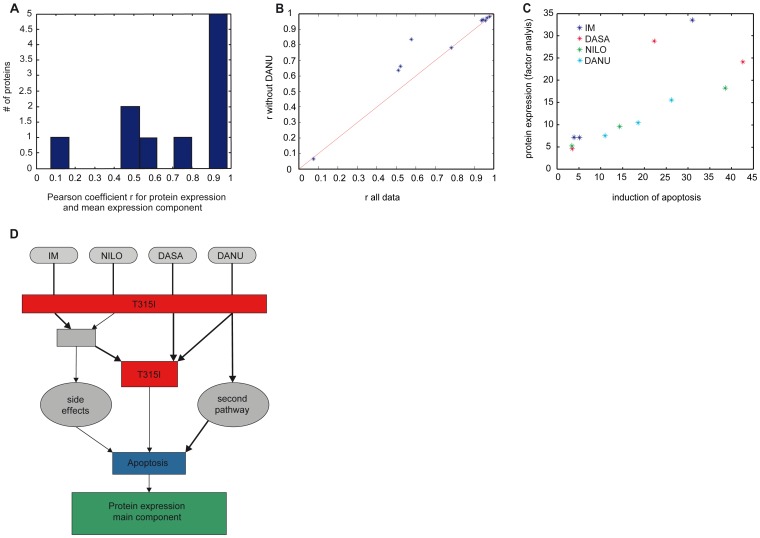
A–D: Meso scale network models for apoptosis induction. (**A**) Distribution of the Pearson coefficient between individual protein expression and the mean component of factor analysis which represents the dominant co-regulation mechanism. (**B**) Shows that the proteins can be decomposed into two groups differing with respect to the impact of DANU. Most proteins show no different co-regulation behaviour if DANU is omitted from the data set, whereas three proteins show a significantly higher degree of co-regulation (increased r value) when DANU is omitted indicating a second mode of action of DANU. (**C**) Shows that with exception of two treatments the mean protein expression, represented by the value of the mean component of the factor analysis (y-axis) is correlated to the observed induction of apoptosis (x-axis) indicating a similar efficacy in apoptosis induction for most drugs. The exceptions indicate that protein expression is induced which does not contribute to apoptosis induction. (**D**) Depicts the meso scale set of pathways, which fits two the observations.

For a subset of 10 proteins induced in all cell lines, factor analysis of the induced expression shifts for all four drugs in all cell lines shows a significant co-regulation of the proteins. Using factor analysis the joint induced expression of the proteins was quantified by the factor FA. The Pearson correlations between the induced protein expressions and the joint induction FA show heterogeneous regulation features (Figure 7A). Five proteins (P63260, P14733, Q61937, P62962, P60710) show linear correlation r>.9, one protein (Q64674) has r = .78. However, the other 4 proteins show significantly lower co-regulation between their expression and FA, indicating that the respective protein expressions are induced by at least two disjoined functional pathways.

In order to identify a differential activation profile of the functional pathways by one of the four TKIs, we performed the same analysis omitting the data from each drug individually. Most significant improvement in co-regulation between FA and the induced protein expressions was found when DANU was omitted (Figure 7B) indicating that DANU induces secondary functional pathways significantly stronger than the other TKIs.

Apparently the expressions of 7 proteins are not affected by specific mechanisms of the MoA of DANU, whereas 3 proteins (Q8R4N0, P00493, Q9R0Q7) show a significant increase of co-regulation with the joint protein expression quantified by FA when DANU is omitted. This result indicates again the existence of a functional pathway to protein expression which is only induced by DANU, but not affected by the other TKIs.

In order to identify co-regulation between induction of apoptosis and overall protein expression, we analyzed the correlation between induced apoptosis rate and the mean component of protein expression discussed above. The results, depicted in Figure 7C, show a surprisingly good correlation between the sum of the induced protein expressions and induced apoptosis. This correlation holds for almost all TKIs and all cell lines, only two outliers (IM in Ba/F3-p210 cells and DASA in Ba/F3-M351T cells) have been found. Detailed analysis of the results depicted in Figure 7C shows that omitting DASA from the analysis results in a reduction of the mean deviation from the linear relationship between the protein induction and apoptosis induction by 18%. We find that the mean protein induction of DASA in the three cell lines is significantly higher than expected by the induction of apoptosis. These results suggest that, in contrast to the other TKI’s, DASA can significantly induce protein expression aside from induction of apoptosis. However, as indicated in Figure 7C, the impact of the drugs on protein expression in relation to induction of apoptosis depends strongly on the type of mutations in the cell lines. Surprisingly we find no significant deviation from the mean protein expression – apoptosis model for DANU, despite, as discussed above, DANU apparently activates a functional pathway which is not induced by the other TKIs.

In summary, the integrated structural analysis of apoptosis induction and protein expression leads to the following findings: i) A group of five proteins (P63260, P14733, Q61937, P62962, P60710) is induced in a highly coherent manner by all TKI and in all cell lines and dominate the main expression component. Hence these proteins may be controlled by secondary response mechanisms which are not specific to the functional pathways of direct drug action. ii) DANU induces a secondary functional pathway which has similar impact on induction of apoptosis than the primary pathway. iii) DASA induces significantly more protein expression in relation to induction of apoptosis than the other drugs. Hence, DASA may show more unspecific effects at the same level of apoptosis induction than the other TKIs. These findings are consistent with a meso scale model for TKI action, which is depicted in Figure 7D, without claiming quantitative interpretation.

## Discussion

Given the lack of selectivity of most TKIs, unexpected side effects of this novel class of drugs are common however differing between compounds. Hence, an easy, high-throughput and unbiased assessment of drug action using a broad panel of drug-induced protein expression data would be of high interest for optimization of drug development. We have developed a modeling approach for reengineering of meso scale networks for assessment of drug action based on a broad panel of proteomics data which are not necessarily involved in the direct mode of action. Although the meso scale networks do not reflect the detailed information with respect to the proteins involved in the MoA, they provide a useful and visual representation of the full systems of pathways involved in the modes of action with a focus on their cross-talk [27], [43], [44]. Our approach does not require a priori information with respect to the modes of action. Nevertheless, it provides critical information for assessment of the drug action in clinical use. Because it is sufficient to unravel the necessary information from secondary effects, the approach allows an unbiased assessment of drug activity. Employing clinically used BCR-ABL inhibitors in different experimental settings, including resistant and non-resistant cell lines, we have demonstrated here how the interaction of multiple MoA can be identified and evaluated using pathway networks on a mesoscopic scale. Furthermore, we show how protein expression data and data about apoptosis induction can be integrated for evaluation of target and non-target drug effects. In contrast to many other studies [45], [46] which are based on gene expression data, our conceptional approach is based on expression data obtained from a global proteomics approach. In comparison to gene expression analysis, the evaluation of the global proteome expression allows for a direct observation of changes in protein expression and even more so in post translational modification of proteins [47]. Using this broad proteomics approach facilitates the identification of unexpected drug effects (which may remain hidden in analysis restricted to pre-selected set of proteins) in an unsupervised approach.

The proteome data described here clearly show, that the different 1^st^, 2^nd^ and 3^rd^ generation TKIs investigated here induce a distinct pattern of protein expression changes which correlates with the drug sensitivity of the individual cell lines studied. These observations are in agreement with the known differences in the inhibitory profile of these TKIs [48], [49], [50], [51]. However, as these studies performed by techinically different chemical or affinitiy purification proteomics approaches which were conducted to identify direct TKI- or BCR-ABL-binding targets rather than effects of TKI inhibition on the protein expression profile of BCR-ABL positive cells, we could not detect any overlap between the proteins identified in these studies and in our proteome screen.

For some of the identified proteins, a connection to BCR-ABL signaling has already been described in previous studies [52]. In particular, the effects of BCR-ABL on actin (which is differentially regulated in our proteome screen) have been linked to the enhanced migration of BCR-ABL positive hematopoietic cells from the bone marrow to the peripheral blood [53]. Heat shock protein 70 (Hsp70) and 90 (Hsp90) are other proteins reported in our proteome analysis that are known targets of BCR-ABL [54], [55]. Interestingly, we identified eIF5A as regulated after treatment with IM, NILO and DASA in Ba/F3-M351T cells. This is in agreement with our recent studies in human K562 cells [52]. Although we identified known targets of BCR-ABL, for most of the described proteins in our proteome screen there are so far no published evidences for a BCR-ABL dependent regulation. Therefore, these data can be used as a basis for further investigation in BCR-ABL signaling. In particular, further investigations in BCR-ABL dependent regulation of TGM2, which is supposed to be involved in apoptosis and adhesion, seems to be worthwhile and are ongoing [56].

In an attempt to demonstrate whether a simplified and coarse-grained approach would gain insights into the differences in of MoA patterns between the compounds studied, we employed meso scale network approach in order to model our protein expression data.

We further analysed the resulting meso scale networks in order to identify similarities and differences between TKIs used for the same clinical indication, i.e. the treatment of Chronic myeloid leukemia. Existing experimental and clinical data indicate that IM, NILO and DASA activate a common MoA, i.e. inhibition of BCR-ABL. In contrast and in addition to inhibition of BCR-ABL, DANU has been shown to inhibit the Aurora family of kinases [38], [42]. Interestingly this phenomenon is indeed reflected by a significantly distinct protein induction profile of DANU as opposed to the other TKIs studied. Even more so, although IM, NILO and DASA clearly exhibit a common dominant MoA in wild-type Ba/F3 cells, they differ significantly with respect to their inhibitory profile when BCR-ABL is mutated in Ba/F3-M351T and Ba/F3-T315I cell lines. Therefore, the profiles of induction of protein expression of IM, NILO and DASA show significant differences, especially if the primary MoA is inhibited by mutations, such that induction of apoptosis is reduced. Whereas NILO appears to be rather specific, DASA induces significant protein expression changes which do not seem to contribute to apoptosis induction in Ba/F3-M351T cell lines. It can only be speculated that based on the specific kinase inhibitory spectrum of DASA, inhibition of members of the SRC-family might contribute to this phenomenom. Future comparisons using alternative combined BCR-ABL/SRC family kinase inhibitors such as Bosutinib might provide additional insight regarding the relevance of SRC inhibition both for induction of apoptosis in leukemic cells as well as for the side effect profile of such TKIs [35], [36], [57]. In contrast to IM, NILO and DASA, DANU shows a very different activation profile. Apparently DANU activates a second pathway which is not affected by the M351T and T315I mutations. This second MoA most likely representing the inhibition of the Aurora family of kinases pathway is almost as efficient in induction of apoptosis as the common primary MoA.

The drawback of the approach in general is the restriction to experimental settings where a clear feed-forward structure of the information flow can be guaranteed. Accordingly, the method can be applied if the MoA of the drug action affects the readout data, here protein expression, but the induced protein expression does not affect the structure of the MoA. If the latter case happens, then validity of the underlying mathematical approach cannot be guaranteed to date. As a consequence, the extension of the method to other drugs than TKI’s can be expected if the drugs act primarily via a well defined set of MoA’s which are not structurally disturbed by the cellular response on the drug itself. Hence, extension to classes of drugs acting by very broad range of targets or inducing a very strong stress response has to be evaluated in future. As unraveling of recursive networks remains an instrinsic challenge for any method explored so far, we see no signficant restriction of the value of the described method as a complementary approach.

Preliminary results indicate that the method can be extended to data sets representing short term drug action, such as phosphoproteomic data [manuscript in preparation]. Future research has to evaluate it`s applicability to data sets reflecting long-term response, such as transcriptomics or epigenetics data. However, more advanced mathematical methods may be able to overcome the abovementioned limitations of the meso-scale approach. Earlier results [Schuppert A, et al.: Method for identifying predictive biomarkers from patient data. Patent WO/2007/07/9875 (2006)] indicate options for extension towards heterogeneous data sets.

In conclusion, our approach led to the identification of direct and indirect drug effects in a well-defined model system and should be amendable to various new drugs. Therefore, combination of broad proteome profiling and meso scale network reengineering provides a versatile tool to map a drug’s direct and indirect target pathways in a single set of experiments. Because of it`s adaptability to other model senarios, this approach should prove valuable at various stages of drug discovery as well as in translational studies of drug action in patient tissues. It represents a powerful method allowing the identification and assessment of multiple MoA using only unbiased protein expression data. Therefore it could contribute significantly to the drug discovery process of compounds acting via complex biological mechanisms.

## Materials and Methods

### Reagents

IM (kindly provided by E. Buchdunger, Novartis, Basel, Switzerland), DANU (kindly provided by Nerviano Medical Sciences, Milan, Italy), NILO (kindly provided by Novartis Pharma, Basel, Switzerland) and DASA (kindly provided by Bristol-Meyers Squibb, New York, NY) were applied according to the different experimental protocols. All tyrosine kinase inhibitors were stored at –20°C as stock solutions (NILO 883µM, DASA 19.76 µM, DANU 10 mM) in DMSO or 50% DMSO (Imatinib 1.7 mM, [DMSO]/H_2_O [1∶1]). Fresh dilutions in complete media were prepared prior to the experiments. The highest concentration of DMSO in cell culture medium was less than or equal to 0.1% and did not have any effect on cell growth.

### Cell Culture Techniques

Ba/F3 and K562 cells were obtained from DSMZ (Bielefeld, Germany). Ba/F3-p210, -T315I and -M351T, and cells were kindly provided by C.L. Sawyers (University of California at Los Angeles) [58], [59]. All cell lines were cultured in RPMI 1640 medium (Gibco-BRL, Invitrogen, Paisley, United Kingdom) containing 10% fetal calf serum (FCS) (Biochrom KG, Berlin, Germany) and for Ba/F3 cells 1 ng/mL recombinant murine interleukin-3 (IL-3) was added. Cells were incubated at 37°C in a humidified atmosphere with 5% CO_2_.

### TKI Treatment for Proteome Analysis

Ba/F3-p210, -T315I and -M351T cells were treated with TKIs (IM 1 µM, NILO 50 nM, DASA 5 nM, DANU 0.4 µM), harvested and washed three times with PBS after 24 h.

### Protein Preparation, Two-dimensional Gel Electrophoresis (2D-PAGE) and Image Analysis

Protein preparation and 2D-PAGE were performed as described previously [52], [60]. In brief, cells were lysed in sample buffer (9 M urea, 4% CHAPS, 0.5% Pharmalyte 3–10 IEF (Amersham Biosciences), 10 µg/mL bromophenol blue) followed by centrifugation at 12 000 *g* for 5 minutes. Samples were applied to linear gradient Immobiline Dry Strip (IPG Strip pH 4–7, 24 cm, Amersham Biosciences, Uppsala, Sweden) by in-gel rehydration. After isoelectric focusing using the Protean IEF cell (Bio-Rad, Hercules, CA) at 10 000 V for approximately 80 kVh, IPG strips were equilibrated for 2×15 minutes in 6 M urea, 4% SDS, 50 mM Tris-HCl, pH 8.8, containing 1% DTT for the first or 4.8% iodoacetamide for the second period of equilibration. Strips were placed on vertical SDS-PAGE gels and overlaid with 0.6% agarose. SDS-PAGE was carried out with the Protean Plus Dodeca Cell (Bio-Rad, Hercules, CA) using 15% SDS-polyacrylamide gels (27 cm×21 cm×1.5 mm). Two-dimensional gels were stained overnight with colloidal Coomassie (0.2% Coomassie Brilliant Blue R250), followed by destaining for 1 day. All experiments were performed in triplicates, revealing comparable results. The 2D-gels were scanned with a GS-800 Calibrated Densitometer (Bio-Rad, Hercules, CA). Images were warped group-wise using Delta2D 3.6 software (Decodon GmbH, Greifswald, Germany). Spot patterns were detected on fused images (gained from all gels) using the average intensity algorithm and retransferred to the original images for 100% matching efficiency. Spot quantification was based on normalized relative spot volume (% volume) as exported from the statistics table of the Delta2D software.

### Protein Identification by Mass Spectrometry

Protein identification was performed as described recently [60], [61]. In brief, trypsin digestion and spotting onto the MALDI-targets were performed in the Ettan Spot Handling Workstation (Amersham-Biosciences, Uppsala, Sweden). The MALDI-TOF measurement of spotted peptide solutions was carried out on a 4800 MALDI TOF/TOF™ Analyzer (Applied Biosystems, Foster City, USA). For protein identification peptide lists were compared with the SwissProt rel.56.1 restricted to murine taxonomy using the Mascot search engine 2.2 (Matrix Science Ltd, London, UK). Peptide mixtures that yielded a mowse score of not less than 55 for SwissProt results were regarded as positive identifications. Additionally to improve probability at least one peptide was sequenced with a significant ion score of above 27.

### Cluster Analysis and Generation of Venn Diagrams

Cluster analysis and the generation of venn diagrams were performed with the statistical language R (http://www.R-project.org). The heat maps from the hierarchical cluster analysis were generated using as a metric Pearson's correlation and the 'average' agglomeration method. For the venn diagrams only proteins were selected showing a p-value> = 95% (from the Lilliefors-test) and a relative expression value over 1.

### Western Blot Analysis

For protein extraction, K562 cells were homogenized on ice in lysis buffer containing 50 mM Tris-HCl, pH 7.5, 150 mM NaCl, 1% NP-40, 0.25% Na-desoxycholate, 5 mM EDTA, 1 mM NaF, 25 mM Na_3_VO_4_, and 0.1 mM PMSF. Lysates were left on ice for 10 minutes, and cellular debris was pelleted at 20 000 *g* for 20 minutes at 4°C. The supernatant was frozen at –80°C. The protein concentration of the lysate was determined with the BCA Protein Assay Kit (Pierce, Rockford, IL). Proteins (20 µg) were separated by 12% SDS-PAGE and transferred onto PVDF membranes with the Bio-Rad Transblot system. After blocking in PBS-Tween/3% wt/vol BSA for 30 minutes, membranes were incubated in primary antibody diluted in PBS-Tween/3% wt/vol BSA. The following primary antisera were used: rabbit anti-Transglutaminase 2 (Abcam, ab421) (1/1000,), mouse monoclonal anti-TGM2 (1/250 ) (Santa Cruz, sc-48387), anti- GAPDH (1/10 000). After washing, membranes were incubated for 1 hour with HRP-conjugated rabbit anti–goat immunoglobulin (1/10 000) or with rabbit anti–mouse immunoglobulin (1/10 000) (both from Amersham Pharmacia Biotech UK, Little Chalfont, Buckinghamshire, United Kingdom), diluted in PBS-Tween/3% wt/vol BSA. After washing, the Pierce ECL Western Blotting Substrate (Thermo Fisher Scientific, Rockford) was used to visualize the secondary antibody.

### Mini-2D Western Blotting Analysis (2D-WB)

2D-WB analysis was performed as previously described [62], [63]. In brief, protein samples were prepared as described for 2D-PAGE. 125 µl solution containing 30 µg of protein were loaded on a linear gradient Immobiline Dry Strip (IPG Strip pH 4–7, 7 cm Amersham Biosciences), followed by rehydration of strips and isoelectric focusing (IEF) using the Protean IEF cell. After equilibration strips were directly loaded onto 15% SDS-polyacrylamide gels, overlayed with 0.6% [w/v] agarose in dH_2_O, and run 3 h at 65 V. Consecutive transfer, blocking, incubation with antibodies and detection steps were carried out as described for the Western blotting experiments, see above.

### Reengineering Meso Scale Models for the MoA of TKIs

In order to unravel insight into the mechanisms of protein expression and induction of apoptosis by TKIs in cells with heterogeneous mutation profiles, we aimed first to establish a meso scale network model for the interaction between the TKIs and mutations. In the second step we developed a model to assess the efficacy of the TKIs, quantified by the induction of apoptosis, in relation to their overall protein induction profile. As outlined above, meso-scale network reconstruction aims to identify the network topology of the relevant pathways as well as their interaction without explicit use of a priori information. In contrast to network reengineering approaches fitting more or less detailed mechanistic models to the data, we intend to provide a proteome-wide, unbiased assessment on the rough structure of the drug induced mechanisms as well as their interaction. Here we are interested in the proteome-scale structure of interactions of the MoA’s of the drugs with the mutations as well as the existence of multiple, drug induced effects. We do not intend to identify the detailed biological mechanisms behind the identified MoA’s and their interaction, which must be done in a complementary follow-up analysis. The mathematical concept has been described recently [41] and is based on the identification of intrinsic correlation structures in the multivariate data sets which are assessed from combinatorial experiments, in contrast to model fitting. In order to exploit the multiple readouts (induced protein expressions and apoptosis), we extended the methods described in [41] by the analysis of the behavior of correlations in wild type and mutated cells. As depicted in Figure S1A–B, the localization of the mutation-induced breakpoint of drug action pathways may be reconstructed by analysis of the correlations of multiple drug-induced readouts between wildtype and mutated cells. Identification of correlation structures means the identification of sets of input variables (TKI’s and mutations) and output variables (protein expression and apoptosis induction) which show a significant degree of correlation in specific sets of the combinatorial inputs. The required decomposition of data with respect to mutual correlations in input and output variables can be performed by clustering. However, due to the small set of combinatorial data, clustering may result in a high degree of instability. However, preliminary analysis showed that the correlations sets showed a hierarchical order of size and correlation. Hence we used the more robust factor analysis (as implemented in Matlab) in an iterative approach, in each step identifying the “leading”correlation sets and analyzing the apparent “outliers” in the following step.

The method as described in [41] [27] allows the identification of non-linear mechanisms, however the identification of the related non-linear correlations using Spearman or Kendall tau instead of Pearson coefficient require large data sets which are not available here. As all correlations appeared to be almost linear, the substitution of non-linear correlation methods by linear correlation analysis was applied significantly improving the stability of the procedure. Combining the multiple linear correlations allowed the identification of the required information with respect to the network topology even from relatively small data sets. So this analysis shows the feasibility proteome wide network reengineering allowing an unbiased assessment of drug induced mechanisms which would not be accessible using detailed network reengineering.

The modeling approach used the combinatorial information with respect to mutation profile and stimulation by TKIs as input variables and the protein expression data as well as the induction of apoptosis as multiple and heterogeneous readouts of the cellular system.

In a first step we identified a model for the pathways and their interaction with mutations describing the induction of protein expression by the TKIs. The analysis was performed using the expression data of all proteins with significant effect of the drugs (inhibition or induction of expression), proteins without significant effect were neglected. If multiple spots were available for one protein, the mean of all spots encoding the protein was used as the effective protein expression. The analysis was performed using Matlab, Statistics toolbox (version R2010b, The Mathworks Inc.).

The protein expressions x_i_(u_j,_ µ_1_) for all available proteins i, induced by one of the four kinase inhibitors u_j_ =  [0,1], j = 1…4, and the mutations µ_l_ l = 1…3 (wt, T351 and T310), were correlated with each other. As described in [41], the meso scale network structure for the mechanism acting between kinase inhibition and protein expression can be established by joint analysis of all pairwise correlations of the protein expressions. If no correlations can be found, the function describing the protein expression in terms of kinase inhibition has to be described using a black-box model as indicated in Figure S2A. However, often factor analysis shows correlations between the protein expressions. Then all protein expressions under all kinase inhibitions can be expressed by a linear factor-model using m (m small) factors γ_k_ depending on the kinase inhibitions u_1_,…,u_4_ and mutations µ_1_,.µ_3_

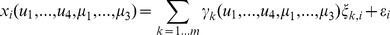
where x_i_(u_1_,…,u_4,_ µ_1_,..µ_3_) describes the expression of protein i depending on the combination of administration of the four kinase inhibitors and mutations. The term ε_i_ represents the deviation of the measured protein expression x_i_ in each experiment (not explicitly annotated) from the value which is predicted by the factor model. As u_1_..u_4_, µ_1_,..µ_3_ are binary variables (kinase inhibitor is administered in the experiment or not), both x_i_ and γ_k_ are functions mapping [0,1]^7^ = >R^1^ for each i and k, respectively. Because of the small size of the data set, which is limited by the set of experiments, identifiability of the number of factors m has been limited. Hence correlations between the protein expressions in all experiments have been analyzed only in 2 and 3 dimensions restricting m to 3. As we found no significant increase of model quality at m = 3 compared to m = 2 we did not analyse higher values of m. Moreover, in order to reduce the dimensionality, the combinations of wt and T351 mutations with IM, DASA and NILO have been analyzed separately from the respective combinations with DANU, as we found a high degree of multi-variate correlations among the proteins in these combinations. Then, in a second step, the deviations induced by T315 mutation and DANU from the model have been analyzed separately. Although this decomposition of the workflow is not necessary, it helped to reduce the effective dimensionality and improving the stability of the respective multivariate correlations and the respective networks.

The factor model indicates that the network representing the mechanism contains m critical nodes (the minimal cut of the network), such that the knock out of these m nodes will be the final blow for the network (Figure S2B). If the factor-model is correct up to random noise for all proteins, then the deviations ε_i_ will show a normal distribution. Alternatively, if the MoA involves two (or more) functional pathways inducing different protein sets each, the kinase inhibitors can each involve either all the functional pathways or a set of pathways specific for each drug (Figure S2C). In the latter case factor analysis will indicate the existence of at least two independent factors as the distribution of the deviations ε_i,_ will show a non-normal distribution. Hence the non-normality of the model deviations ε_i_ provides a mean for discrimination between models of type 1b) and 1c). In the latter case the protein groups have to be classified using regression clustering methods.

### Integration of Protein Expression Data and Apoptosis Induction

In the second step we assessed the efficiency of the drugs induced apoptosis compared to the drug induced protein expression. We aimed to develop a meso scale model describing only the dominant routes from drug action to both induction of apoptosis and protein expression as well as their interaction with mutations. In contrast to the analysis described above, we had to integrate heterogeneous readouts, namely apoptosis and protein expressions into a meso scale model.

To identify the meso scale network which represents the drug induced protein expressions as well as induction of apoptosis in Ba/F3-p210-, M351T and -T315I cell lines, we focused the analysis on the subset of 10 proteins showing differential expression in all three cell lines.

To establish the model for apoptosis induction, the multi-variate correlations between the protein expressions have been assessed using factor analysis, combined with the analysis of the distributions of the residues. The mechanism of induction of protein expression and apoptosis by TKIs may differ in detail between the drugs, although the principal MoA, namely the inhibition of BCR-ABL, is the same. Hence the data represent a convolution of the main, common MoA and drug specific mechanisms, which are hard to reconstruct in a direct, one step approach. Hence we choose a two-step modeling workflow. In a first step of approximation, factor analysis was used for the characterization of the common, dominant mechanism of induction of protein expression together with apoptosis for all TKIs. As factor analysis tends to neglect apparent “outliers” in the data, it is superior to linear models like PLS in order to quantify the main contributions of the TKIs and the mutations to the dominant mechanism without mixing drug-specific mechanisms with the common mechanism.

In a second step the specific properties of the TKIs have been assessed by analysis of the TKI- and mutation specific deviations from the dominant factor model identified in the first step. In this approach the deviations from the model describing the dominant mechanism are not interpreted as noise. They serve as a filter which allows to decompose the data with respect to information induced by an overall dominant mechanism (which is not specific to the TKI’s) and the information which is induced by mechanisms specific to the respective TKI.

## Supporting Information

Figure S1 **A–B:** **These figures show the concept of analysis of correlations of drug-induced expression shifts for large protein sets between wildtype and mutated cells in order to identify the localisation of the mutation-induced breakpoint.** In **(A)** the mutation affects the pathways towards all proteins equally by breaking the pathway upstream of the first bifurcation node (blue bullet). In contrast, **(B)** shows that a breakpoint downstream the first bifurcation node affects the structure of the correlations of the drug-induced expression among the set of proteins. Hence analysis of the structure of correlations reveals infromation with respect to the localisation of the mutation-induced breakpoint.
(TIF)

Figure S2 **A–C: Schematic representation of meso scale pathways structure.** **(A)** Network structure if no correlations between the 4 drugs can be found, **(B)** if critical meso scale nodes can be described and **(C)** if different drugs acted on different nodes.
(TIF)

Table S1 **Proteins significantly regulated in Ba/F3-p210 cells.** Lists of Proteins that were significantly regulated in each of the subsets (IM, NILO, DASA and DANU). The relative expression values compared to the average expression values of control samples (DMSO) are presented.
(DOC)

Table S2 **Proteins significantly regulated in Ba/F3-M351T cells.** Lists of Proteins that were significantly regulated in each of the subsets (IM, NILO, DASA and DANU). The relative expression values compared to the average expression values of control samples (DMSO) are presented.
(DOC)

Table S3 **Proteins significantly regulated in Ba/F3-T315I cells.** Lists of Proteins that were significantly regulated in each of the subsets (IM, NILO, DASA and DANU). The relative expression values compared to the average expression values of control samples (DMSO) are presented.
(DOC)

Table S4 **Proteins affected by DANU in Ba/F3-p210 cells analyzed by regression clustering.**
(DOC)
