# Supplementary figures and images for: Combination of a Proteomics Approach and Reengineering of Meso Scale Network Models for Prediction of Mode-of-Action for Tyrosine Kinase Inhibitors
Source: PLoS One. 2013 Jan 9;8(1):e53668. doi: 10.1371/journal.pone.0053668 (PMC3541187; doi:10.1371/journal.pone.0053668)

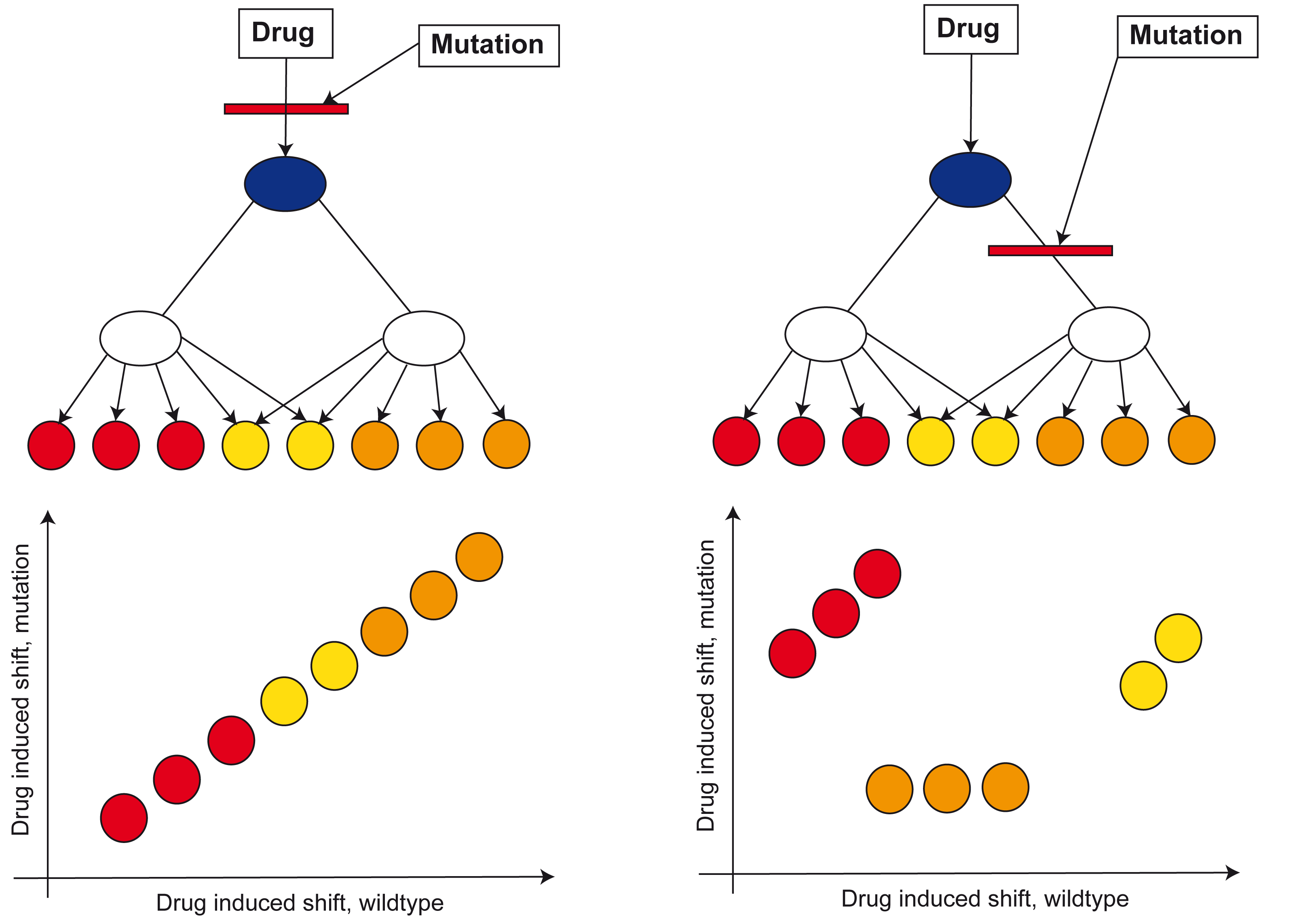

Supplement: Figure S1 — A–B: These figures show the concept of analysis of correlations of drug-induced expression shifts for large protein sets between wildtype and mutated cells in order to identify the localisation of the mutation-induced breakpoint. In (A) the mutation affects the pathways towards all proteins equally by breaking the pathway upstream of the first bifurcation node (blue bullet). In contrast, (B) shows that a breakpoint downstream the first bifurcation node affects the structure of the correlations of the drug-induced expression among the set of proteins. Hence analysis of the structure of correlations reveals infromation with respect to the localisation of the mutation-induced breakpoint. (TIF) [file pone.0053668.s001.tif]

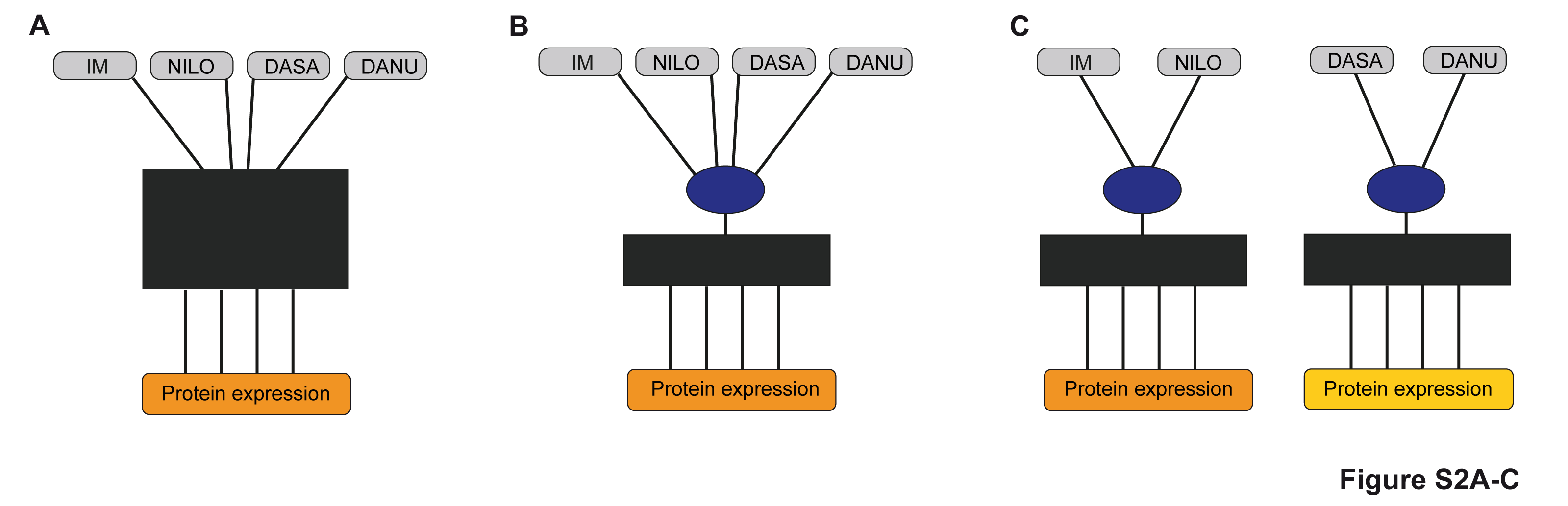

Supplement: Figure S2 — A–C: Schematic representation of meso scale pathways structure. (A) Network structure if no correlations between the 4 drugs can be found, (B) if critical meso scale nodes can be described and (C) if different drugs acted on different nodes. (TIF) [file pone.0053668.s002.tif]
