# Supplementary material for: Combination of a Proteomics Approach and Reengineering of Meso Scale Network Models for Prediction of Mode-of-Action for Tyrosine Kinase Inhibitors
Source: PLoS One. 2013 Jan 9;8(1):e53668. doi: 10.1371/journal.pone.0053668 (PMC3541187; doi:10.1371/journal.pone.0053668)
Supplement: Table S1 — Proteins significantly regulated in Ba/F3-p210 cells. Lists of Proteins that were significantly regulated in each of the subsets (IM, NILO, DASA and DANU). The relative expression values compared to the average expression values of control samples (DMSO) are presented. (DOC) [file pone.0053668.s003.doc]

| **Spot** | **Swiss Prot ID** | **Protein name** | **IM** | **NILO** | **DASA** | **DANU** |
| --- | --- | --- | --- | --- | --- | --- |
| 2 | Q8CGK3 | Lon protease homolog. mitochondrial | **2.85** | 1.72 | 1.58 | 1.03 |
| 3 | Q8VDD5 | Myosin heavy chain. non-muscle IIa | **2.37** | **2.08** | **2.72** | 0.76 |
| 4 | Q8VDD5 | Myosin heavy chain. non-muscle IIa | **2.64** | **2.09** | **2.85** | 0.82 |
| 7 | Q64674 | Spermidine synthase | **4.99** | 1.88 | **4.29** | 1.05 |
| 11 | P07901 | HSP 90-alpha | **3.58** | 1.81 | **2.51** | 1.50 |
| 12 | O35226 | 26S proteasome non-ATPase regulatory subunit 4 | 0.60 | 0.80 | **0.41** | 0.60 |
| 13 | P05213 | Tubulin alpha-1B chain | **5.63** | **4.05** | **4.50** | 1.69 |
| 16 | Q7TMK9 | Heterogeneous nuclear ribonucleoprotein Q | **2.22** | 1.14 | 1.37 | 0.82 |
| 17 | P61979 | Heterogeneous nuclear ribonucleoprotein K | **4.70** | **4.90** | **4.06** | 1.56 |
| 18 | Q61316 | Heat shock 70 kDa protein 4 | **2.58** | 1.71 | **2.04** | **3.77** |
| 20 | Q7TMK9 | Heterogeneous nuclear ribonucleoprotein Q | **2.51** | 1.09 | 1.52 | 0.88 |
| 21 | P63260 | Actin. cytoplasmic 2 | **3.27** | 1.53 | **3.13** | 1.15 |
| 26 | P14869 | 60S acidic ribosomal protein P0 | **4.38** | **4.31** | **3.92** | 1.01 |
| 30 | P21981 | Protein-glutamine gamma-glutamyltransferase 2 | **3.57** | 1.65 | **2.23** | 1.33 |
| 31 | Q6PDM2 | Splicing factor. arginine/serine-rich 1 | **0.43** | 0.85 | 0.64 | 0.94 |
| 32 | Q64674 | Spermidine synthase | **0.28** | 0.85 | 0.68 | 0.73 |
| 34 | Q64674 | Spermidine synthase | **3.79** | 1.78 | **3.69** | 1.83 |
| 36 | Q8R4N0 | Citrate lyase subunit beta-like protein | 1.57 | 1.47 | 1.23 | **0.21** |
| 38 | P63260 | Actin. cytoplasmic 2 | **2.47** | 1.36 | **2.23** | 1.01 |
| 39 | P63260 | Actin. cytoplasmic 2 | **4.32** | **2.59** | **3.94** | 0.80 |
| 41 | P63260 | Actin. cytoplasmic 2 | **6.12** | **2.29** | **4.95** | 1.07 |
| 42 | P80314 | T-complex protein 1 subunit beta | **3.05** | **2.08** | 1.79 | 1.27 |
| 43 | P63260 | Actin. cytoplasmic 2 | **3.35** | **2.00** | **4.77** | 1.00 |
| 45 | Q9CQ65 | S-methyl-5'-thioadenosine phosphorylase | **3.96** | **2.43** | **3.50** | 0.96 |
| 47 | Q60817 | Nascent polypeptide-associated complex subunit alpha | **2.08** | 0.64 | 1.40 | 1.31 |
| 48 | O70251 | Elongation factor 1-beta | 1.83 | **2.43** | **2.06** | 1.18 |
| 49 | P63073 | Eukaryotic translation initiation factor 4E | 1.03 | **3.28** | 1.01 | 0.60 |
| 51 | P14733 | Lamin-B1 | **7.35** | **10.72** | **3.37** | **4.64** |
| 53 | P60335 | Poly(rC)-binding protein 1 | **3.06** | **2.12** | **2.95** | 1.11 |
| 54 | P14733 | Lamin-B1 | **8.04** | 1.42 | **6.13** | **2.60** |
| 55 | P00493 | Hypoxanthine-guanine phosphoribosyltransferase | 1.11 | 0.96 | 1.26 | **2.41** |
| 56 | Q61937 | Nucleophosmin | **12.10** | 1.55 | **6.65** | **2.52** |
| 57 | P63260 | Actin. cytoplasmic 2 | **5.74** | **2.73** | **5.04** | 1.29 |
| 59 | Q61937 | Nucleophosmin | **5.40** | 1.28 | **5.76** | 1.28 |
| 62 | Q61937 | Nucleophosmin | **4.62** | 1.57 | 2.52 | 1.13 |
| 63 | P84104 | Splicing factor. arginine/serine-rich 3 | **0.36** | 1.00 | 0.61 | 0.87 |
| 66 | Q9ESP1 | Stromal cell-derived factor 2-like protein 1 | 0.54 | 1.27 | 0.84 | **0.16** |
| 69 | P09405 | Nucleolin | **5.21** | **3.40** | **4.85** | 1.71 |
| 71 | Q9R0Q7 | Prostaglandin E synthase 3 | **0.35** | 0.61 | **0.43** | 0.81 |
| 72 | Q9R0Q7 | Prostaglandin E synthase 3 | **0.34** | 0.58 | **0.46** | 0.87 |
| 75 | P33316 | Deoxyuridine 5'-triphosphate nucleotidohydrolase | **0.48** | 1.12 | **0.49** | 1.30 |
| 79 | Q01768 | Nucleoside diphosphate kinase B | **0.07** | **0.17** | 1.03 | **0.11** |
| 80 | P63242 | Eukaryotic translation initiation factor 5A-1 | **0.41** | **0.47** | **0.38** | 0.67 |
| 81 | Q3UAJ1 | Peptidyl-prolyl cis-trans isomerase | **0.37** | **0.35** | 0.76 | 0.85 |
| 82 | P63242 | Eukaryotic translation initiation factor 5A-1 | **6.60** | **5.16** | **5.69** | 1.79 |
| 87 | P63260 | Actin. cytoplasmic 2 | **3.02** | **3.06** | **2.60** | 1.16 |
| 88 | Q3U804 | Actb. Actin beta | **2.36** | **2.58** | 2.18 | 1.24 |
| 92 | Q8CGP1 | Histone H2B type 1-K | **0.16** | **0.27** | **0.18** | **0.49** |
| 94 | P63323 | 40S ribosomal protein S12 | **0.07** | 1.16 | 0.98 | 1.43 |
| 95 | Q3THW5 | Histone H2AV | **2.51** | 1.75 | 1.94 | 1.11 |
| 100 | P62962 | Profilin-1 | **8.64** | **5.95** | **5.63** | **2.32** |
| 104 | P60710 | Actin, cytoplasmic 1 | **4.70** | 1.96 | **3.61** | 1.10 |

**Table S1:** Proteins significantly regulated in Ba/F3-p210 cells. List of proteins that were significantly regulated in each of the subsets (IM, NILO, DASA and DANU). The relative expression values compared to the average expression values of control samples (DMSO) are presented.
