# Supplementary material for: Combination of a Proteomics Approach and Reengineering of Meso Scale Network Models for Prediction of Mode-of-Action for Tyrosine Kinase Inhibitors
Source: PLoS One. 2013 Jan 9;8(1):e53668. doi: 10.1371/journal.pone.0053668 (PMC3541187; doi:10.1371/journal.pone.0053668)
Supplement: Table S2 — Proteins significantly regulated in Ba/F3-M351T cells. Lists of Proteins that were significantly regulated in each of the subsets (IM, NILO, DASA and DANU). The relative expression values compared to the average expression values of control samples (DMSO) are presented. (DOC) [file pone.0053668.s004.doc]

| **Spot** | **Swiss Prot ID** | **Protein name** | **IM** | **NILO** | **DASA** | **DANU** |
| --- | --- | --- | --- | --- | --- | --- |
| 3 | Q8VDD5 | Myosin heavy chain. non-muscle IIa | 1.20 | 0.64 | **2.52** | 0.82 |
| 7 | Q64674 | Spermidine synthase | 1.71 | 1.68 | **6.85** | **2.12** |
| 10 | P05213 | Tubulin alpha-1B chain | 1.34 | 1.24 | **2.66** | 0.85 |
| 17 | P61979 | Heterogeneous nuclear ribonucleoprotein K | 0.93 | 1.39 | **3.35** | 1.35 |
| 20 | Q7TMK9 | Heterogeneous nuclear ribonucleoprotein Q | 1.12 | 0.88 | **2.51** | 1.08 |
| 21 | P63260 | Actin. cytoplasmic 2 | 1.09 | 0.79 | **2.79** | 0.89 |
| 26 | P14869 | 60S acidic ribosomal protein P0 | 0.81 | 1.57 | **3.45** | 0.53 |
| 34 | Q64674 | Spermidine synthase | 1.00 | 1.59 | **3.55** | 1.18 |
| 36 | Q8R4N0 | Citrate lyase subunit beta-like protein. mitochondrial | 0.70 | 0.64 | 1.11 | **0.19** |
| 38 | P63260 | Actin. cytoplasmic 2 | 1.85 | 1.46 | **2.08** | **2.07** |
| 39 | P63260 | Actin. cytoplasmic 2 | 0.95 | 1.21 | **3.00** | 1.02 |
| 41 | P63260 | Actin. cytoplasmic 2 | 1.76 | 1.46 | **5.82** | 1.02 |
| 42 | P80314 | T-complex protein 1 subunit beta | 1.82 | **2.00** | **3.58** | 1.33 |
| 43 | P63260 | Actin. cytoplasmic 2 | 1.51 | 1.90 | **4.35** | 1.25 |
| 44 | O35381 | Acidic leucine-rich nuclear phosphoprotein 32 family member A | 0.66 | 1.54 | 0.85 | **0.34** |
| 45 | Q9CQ65 | S-methyl-5'-thioadenosine phosphorylase | 1.05 | 1.21 | **3.02** | 1.17 |
| 47 | Q60817 | Nascent polypeptide-associated complex subunit alpha | 1.31 | 1.03 | **2.75** | 1.05 |
| 53 | P60335 | Poly(rC)-binding protein 1 | 0.63 | 1.40 | **2.36** | 1.36 |
| 54 | P14733 | Lamin-B1 | 1.18 | **2.62** | **6.74** | 0.92 |
| 55 | P00493 | Hypoxanthine-guanine phosphoribosyltransferase | 0.59 | 1.10 | **2.95** | 0.45 |
| 56 | Q61937 | Nucleophosmin | 1.12 | **2.03** | **7.44** | 1.05 |
| 57 | P63260 | Actin. cytoplasmic 2 | 0.94 | 1.05 | **2.82** | 1.03 |
| 59 | Q61937 | Nucleophosmin | 0.88 | **2.40** | **4.96** | 1.07 |
| 62 | Q61937 | Nucleophosmin | 0.97 | 1.12 | **4.09** | 0.71 |
| 66 | Q9ESP1 | Stromal cell-derived factor 2-like protein 1 [Precursor] | 0.81 | **0.21** | 1.22 | **0.18** |
| 68 | Q3THE2 | Myosin regulatory light chain 2-B. smooth muscle isoform | **0.48** | 0.56 | 1.08 | 0.61 |
| 69 | P09405 | Nucleolin | 0.66 | 1.76 | **4.21** | 1.04 |
| 70 | Q9D898 | Actin-related protein 2/3 complex subunit 5-like protein | 0.57 | **0.30** | 0.63 | 0.55 |
| 71 | Q9R0Q7 | Prostaglandin E synthase 3 | 0.64 | 0.78 | 0.83 | **0.45** |
| 77 | P60710 | Actin. cytoplasmic 1 | 1.69 | 1.39 | **2.79** | **2.30** |
| 79 | Q01768 | Nucleoside diphosphate kinase B | 0.62 | 0.97 | 0.94 | **0.04** |
| 82 | P63242 | Eukaryotic translation initiation factor 5A-1 | 0.99 | 1.82 | **5.05** | 1.04 |
| 100 | P62962 | Profilin-1 | 1.61 | 1.82 | **6.79** | 1.84 |
| 104 | P60710 | Actin. cytoplasmic 1 | 0.85 | 1.10 | **3.57** | 1.26 |

**Table S2:** Proteins significantly regulated in Ba/F3-M351T cells. List of proteins that were significantly regulated in each of the subsets (IM, NILO, DASA and DANU). The relative expression values compared to the average expression values of control samples (DMSO) are presented.
