# Supplementary material for: Combination of a Proteomics Approach and Reengineering of Meso Scale Network Models for Prediction of Mode-of-Action for Tyrosine Kinase Inhibitors
Source: PLoS One. 2013 Jan 9;8(1):e53668. doi: 10.1371/journal.pone.0053668 (PMC3541187; doi:10.1371/journal.pone.0053668)
Supplement: Table S3 — Proteins significantly regulated in Ba/F3-T315I cells. Lists of Proteins that were significantly regulated in each of the subsets (IM, NILO, DASA and DANU). The relative expression values compared to the average expression values of control samples (DMSO) are presented. (DOC) [file pone.0053668.s005.doc]

| **Spot** | **Swiss Prot ID** | **Protein name** | **IM** | **NILO** | **DASA** | **DANU** |
| --- | --- | --- | --- | --- | --- | --- |
| 7 | Q64674 | Spermidine synthase | **2.57** | 1.56 | 1.06 | **2.92** |
| 33 | Q8R4N0 | Citrate lyase subunit beta-like protein | **0.30** | 0.48 | 0.77 | **3.13** |
| 34 | Q64674 | Spermidine synthase | 1.55 | 0.89 | 1.72 | **2.59** |
| 36 | Q8R4N0 | Citrate lyase subunit beta-like protein | **0.15** | 0.41 | 0.74 | **2.00** |
| 39 | P63260 | Actin. cytoplasmic 2 | 1.13 | **0.32** | 0.94 | 1.05 |
| 41 | P63260 | Actin. cytoplasmic 2 | 1.94 | 1.32 | 0.69 | **2.57** |
| 43 | P63260 | Actin. cytoplasmic 2 | 1.35 | 1.15 | 0.84 | **2.32** |
| 49 | P63073 | Eukaryotic translation initiation factor 4E | 0.93 | **12.02** | 0.70 | **0.42** |
| 51 | P14733 | Lamin-B1 | 0.57 | 0.95 | **0.38** | **2.64** |
| 55 | P00493 | Hypoxanthine-guanine phosphoribosyltransferase | **0.46** | **0.42** | 0.88 | 1.39 |
| 56 | Q61937 | Nucleophosmin | 0.98 | 0.77 | 0.60 | **2.80** |
| 62 | Q61937 | Nucleophosmin | 0.72 | 1.07 | **0.40** | 2.24 |
| 72 | Q9R0Q7 | Prostaglandin E synthase 3 | 0.64 | 1.15 | 0.98 | **0.47** |
| 75 | P33316 | Deoxyuridine 5'-triphosphate nucleotidohydrolase | **0.18** | 1.12 | 0.96 | 0.71 |
| 77 | P60710 | Actin. cytoplasmic 1 | 1.44 | 0.98 | 0.99 | **2.44** |
| 79 | Q01768 | Nucleoside diphosphate kinase B | **0.21** | **0.28** | 0.96 | **2.27** |
| 81 | Q3UAJ1 | Peptidyl-prolyl cis-trans isomerase | 1.11 | 0.75 | 1.08 | **2.26** |
| 88 | Q3U804 | Actb. Actin beta | 0.76 | **2.14** | 0.60 | 1.71 |
| 94 | P63323 | 40S ribosomal protein S12 | **0.36** | 0.69 | 0.95 | 1.00 |
| 97 | Q9EST5 | Acidic leucine-rich nuclear phosphoprotein 32 family member B | **2.12** | 1.41 | 0.77 | 0.95 |
| 100 | P62962 | Profilin-1 | 1.78 | 1.18 | 1.08 | **3.62** |

**Table S3:** Proteins significantly regulated in Ba/F3-T315I cells. List of proteins that were significantly regulated in each of the subsets (IM, NILO, DASA and DANU). The relative expression values compared to the average expression values of control samples (DMSO) are presented.
