# Supplementary material for: Combination of a Proteomics Approach and Reengineering of Meso Scale Network Models for Prediction of Mode-of-Action for Tyrosine Kinase Inhibitors
Source: PLoS One. 2013 Jan 9;8(1):e53668. doi: 10.1371/journal.pone.0053668 (PMC3541187; doi:10.1371/journal.pone.0053668)
Supplement: Table S4 — Proteins affected by DANU in Ba/F3-p210 cells analyzed by regression clustering. (DOC) [file pone.0053668.s006.doc]

| **Swiss-Prot ID** | **Protein name** |
| --- | --- |
| **Group 2** | |
| O35226 | 26S proteasome non-ATPase regulatory subunit 4 |
| Q61316 | Heat shock 70 kDa protein 4 |
| Q6PDM2 | Splicing factor, arginine/serine-rich 1 |
| P00493 | Hypoxanthine-guanine phosphoribosyltransferase |
| P84104 | Splicing factor, arginine/serine-rich 3 |
| Q9R0Q7 | Prostaglandin E synthase 3 |
| P33316 | Deoxyuridine 5'-triphosphate nucleotidohydrolase, mitochondrial |
| Q3UAJ1 | Peptidyl-prolyl cis-trans isomerase |
| Q8CGP1 | Histone H2B type 1-K |
| P63323 | 40S ribosomal protein S12 |
|  | |
| **Group 1** | |
| Q8CGK3 | Lon protease homolog, mitochondrial [Precursor] |
| Q8VDD5 | Myosin heavy chain, non-muscle IIa |
| Q64674 | Spermidine synthase |
| P07901 | Heat shock protein HSP 90-alpha |
| P05213 | Tubulin alpha-1B chain |
| Q7TMK9 | Heterogeneous nuclear ribonucleoprotein Q |
| P61979 | Heterogeneous nuclear ribonucleoprotein K |
| P63260 | Actin, cytoplasmic 2 |
| P14869 | 60S acidic ribosomal protein P0 |
| P21981 | Protein-glutamine gamma-glutamyltransferase 2 |
| Q8R4N0 | Citrate lyase subunit beta-like protein, mitochondrial |
| P80314 | T-complex protein 1 subunit beta |
| Q9CQ65 | S-methyl-5'-thioadenosine phosphorylase |
| Q60817 | Nascent polypeptide-associated complex subunit alpha |
| O70251 | Elongation factor 1-beta |
| P63073 | Eukaryotic translation initiation factor 4E |
| P14733 | Lamin-B1 |
| P60335 | Poly(rC)-binding protein 1 |
| Q61937 | Nucleophosmin |
| Q9ESP1 | Stromal cell-derived factor 2-like protein 1 [Precursor] |
| P09405 | Nucleolin |
| Q01768 | Nucleoside diphosphate kinase B |
| P63242 | Eukaryotic translation initiation factor 5A-1 |
| Q3U804 | Actin beta |
| Q3THW5 | Histone H2AV |
| P62962 | Profilin-1 |
| P60710 | Actin, cytoplasmic 1 |

**Table S4:** Proteins affected by DANU in Ba/F3-p210 cells analyzed by regression clustering.
